# Investigation of the safety and feasibility of AAV1/SERCA2a gene transfer in patients with chronic heart failure supported with a left ventricular assist device – the SERCA-LVAD TRIAL

**DOI:** 10.1038/s41434-020-0171-7

**Published:** 2020-07-15

**Authors:** A. R. Lyon, D. Babalis, A. C. Morley-Smith, M. Hedger, A. Suarez Barrientos, G. Foldes, L. S. Couch, R. A. Chowdhury, K. N. Tzortzis, N. S. Peters, E. A. Rog-Zielinska, H-Y Yang, S. Welch, C. T. Bowles, S. Rahman Haley, A. R. Bell, A. Rice, T. Sasikaran, N. A. Johnson, E. Falaschetti, J. Parameshwar, C. Lewis, S. Tsui, A. Simon, J. Pepper, J. J. Rudy, K. M. Zsebo, K. T. Macleod, C. M. Terracciano, R. J. Hajjar, N. Banner, S. E. Harding

**Affiliations:** 1 National Heart and Lung Institute, Imperial College London, London, UK; 2 NIHR Cardiovascular Biomedical Research Unit, Royal Brompton and Harefield Hospitals NHS Trust, London, UK; 3 Imperial Clinical Trials Unit (ICTU), School of Public Health, Imperial College London, London, UK; 4 Institute for Experimental Cardiovascular Medicine, University Heart Center, Medical Center, University of Freiburg, Freiburg, Germany; 5 Department of Histopathology, Royal Brompton and Harefield Hospitals NHS Trust, Freiburg, Germany; 6 Royal Papworth Hospital NHS Trust, Cambridge, UK; 7 Celladon Corporation, San Diego, CA USA; 8 Phospholamban Foundation, Amsterdam, Netherlands

**Keywords:** Cardiovascular diseases, Gene therapy

## Abstract

The SERCA-LVAD trial was a phase 2a trial assessing the safety and feasibility of delivering an adeno-associated vector 1 carrying the cardiac isoform of the sarcoplasmic reticulum calcium ATPase (AAV1/SERCA2a) to adult chronic heart failure patients implanted with a left ventricular assist device. The SERCA-LVAD trial was one of a program of AAV1/SERCA2a cardiac gene therapy trials including CUPID1, CUPID 2 and AGENT trials. Enroled subjects were randomised to receive a single intracoronary infusion of 1 × 10^13^ DNase-resistant AAV1/SERCA2a particles or a placebo solution in a double-blinded design, stratified by presence of neutralising antibodies to AAV. Elective endomyocardial biopsy was performed at 6 months unless the subject had undergone cardiac transplantation, with myocardial samples assessed for the presence of exogenous viral DNA from the treatment vector. Safety assessments including ELISPOT were serially performed. Although designed as a 24 subject trial, recruitment was stopped after five subjects had been randomised and received infusion due to the neutral result from the CUPID 2 trial. Here we describe the results from the 5 patients at 3 years follow up, which confirmed that viral DNA was delivered to the failing human heart in 2 patients receiving gene therapy with vector detectable at follow up endomyocardial biopsy or cardiac transplantation. Absolute levels of detectable transgene DNA were low, and no functional benefit was observed. There were no safety concerns in this small cohort. This trial identified some of the challenges of performing gene therapy trials in this LVAD patient cohort which may help guide future trial design.

## Introduction

Sarcoplasmic reticulum calcium ATPase 2a (SERCA2a) gene therapy is being developed as a potential new treatment for chronic heart failure (HF). SERCA2a gene therapy aims to deliver effective levels of SERCA2a cDNA to the failing myocardium in order to increase production of functional SERCA2a protein and enzyme activity. Restoration of SERCA2a protein levels and activity has recovered left ventricular function in a range of preclinical small and large animal HF models [1–6], with an additional antiarrhythmic benefit [7, 8], and also improved contractile function in isolated failing human cardiomyocytes [9].

There was a program of clinical trials including CUPID 1, CUPID 2, AGENT-HF and the present trial, SERCA-LVAD, aiming to understand the safety, feasibility and then efficacy, of cardiac SERCA2a gene therapy [10–13]. The initial CUPID1 trial delivering SERCA2a gene therapy via intracoronary infusion of an adeno-associated viral vector (AAV) encoding the SERCA2a gene, under regulation of a cytomegalovirus promoter, to adult HF patients was published in 2011 [10]. This showed the procedure safety in a dose-response study and a potential efficacy signal in the subgroup receiving 1 × 10^13^ DNase-resistant particles. The safety and improvements were maintained at 3 years follow up [11]. CUPID2, a phase 2b randomised, placebo-controlled trial, recruited 250 patients and was powered for efficacy, but showed no benefit of this dose (although made by a different manufacturing process compared with CUPID1) in patients with advanced HF [12]. The AGENT-HF and SERCA-LVAD trials were terminated prematurely when the CUPID2 trial reported neutral results. Both AGENT-HF, which was specifically investigating ventricular remodelling and recruited 9 patients [13], and the SERCA-LVAD trial used the same dose and batch of AAV1/SERCA2a as CUPID2.

Left ventricular assist devices (LVADs) are a mechanical circulatory support treatment for patients with advanced HF and are typically used as a bridge-to-heart transplantation or as a destination therapy [14]. There has been increasing LVAD use in the last 15 years, leading to a growing population of HF patients living with LVADs. Long term LVAD treatment is associated with a number of potential complications, including infection, stroke, haemorrhage, pump thrombosis, acquired aortic valve regurgitation, right HF and (now rarely) mechanical failure. Urgent LVAD implantation for acute cardiac failure (e.g., acute myocarditis, peripartum cardiomyopathy) may facilitate ventricular recovery, weaning and explantation (bridge-to-recovery). Efforts to recover the function of failing myocardium in patients with chronic HF have been more challenging. There is evidence that patients who successfully recovered ventricular function during LVAD support had higher sarcoplasmic reticulum (SR) calcium stores, and faster relaxation of myocytes biopsied, compared with patients who failed to recover and myocytes from end-stage failing hearts pre-LVAD or transplant [15]. Whether these changes were causative or associative is unknown, but these observations raise the hypothesis that improving myocardial calcium physiology via restoring SERCA2a activity and SR calcium stores could improve ventricular function and possibly recover cardiac function to a level where LVAD support could be weaned.

The SERCA-LVAD trial was designed to address the safety and feasibility of SERCA2a gene therapy delivered using an AAV vector to adult patients with chronic HF and LVAD support. The specific aims of the trial were to assess safety of SERCA2a gene therapy in this new patient population and the magnitude of viral gene transfer to the human failing myocardium in a systematic manner. It had also been intended to investigate the influence of prior circulating neutralising antibodies (NAbs) to AAV1 upon myocardial gene transfer, although the recruitment of only one NAb positive patient meant that this question went unanswered.

This trial was initiated in 2014 but following the announcement of the neutral result of CUPID2 trial in April 2015, the SERCA-LVAD trial steering committee suspended recruitment after reviewing the risk:benefit balance for study subjects. Here we present the 3 year follow up results from the 5 subjects who were enroled, randomised and received investigational medicinal product (IMP) infusion in the SERCA-LVAD trial.

## Methods

### Study overview

The SERCA-LVAD trial was a phase 2a randomised, double-blind, placebo controlled trial assessing the safety and feasibility of a single intracoronary infusion of AAV1/SERCA2a gene therapy in adults with chronic HF supported with a LVAD. Subjects were randomised in 2:1 ratio to SERCA2a gene therapy or placebo infusion, with randomisation stratified and based on circulating anti-AAV1 NAb status (presence or absence) in a 1:1 ratio. All patients were scheduled for a left ventricular (LV) endomyocardial biopsy (EMB) at 6 months, unless they underwent cardiac transplantation in the first 6 months following trial infusion. The primary endpoint was safety at 6 months and the key secondary endpoint was presence of vector-specific transgene measured by polymerase chain reaction (PCR) in LV myocardial samples from EMB or cardiac transplantation. Long term safety will be recorded for 10 years as part of routine clinical practice, in accordance with the requirements of the Advanced Therapies Medicinal Products Regulation. The trial was registered (EudraCT number: 2007-002809-48; ClinicalTrials.gov identifier: NCT00534703), sponsored by Imperial College London. Ethical approval for the study was granted by the UK Gene Therapy Advisory Committee of the National Research Ethics Service and approval was also obtained from the Medicines and Healthcare Products Regulatory Agency. The trial was conducted according to the principles of the Declaration of Helsinki and the EU Clinical Trial Directive, and all human tissue was collected under appropriate ethical approval. All patients provided written informed consent.

### Trial oversight

We established a Trial Steering Committee (TSC) to oversee study conduct and an independent Data Safety and Monitoring Committee to review safety. The trial was managed by the Imperial Clinical Trials Unit. We used the InForm Integrated Trial Management system that included a web-based electronic case record form, built-in validation rules, adverse event reporting, and a complete audit trail. Participating NHS sites received initiation, routine monitoring, and closeout visits.

### Patient population

Eligible patients were adults (18–70 years) who had an LVAD implanted for chronic HF with reduced ejection fraction (HFrEF), were clinically stable in the opinion of the clinical team looking after the patient (e.g., no chronic infection, no LVAD-associated complications) and able to provide informed consent (see detailed inclusion and exclusion criteria in Supplementary Data Table S1). Chronic HF was defined as a diagnosis of HFrEF and treatment with guideline-based treatment for HFrEF for a minimum of 6 months prior to LVAD implantation, or if less than 6 months between diagnosis and LVAD implantation then a total of 6 months including LVAD support and without evidence of recovery.

Exclusion criteria included biventricular VAD support, acute infection within 48 h prior to administration of IMP, previous LVAD-associated thrombosis with the current LVAD, a persistently raised lactate dehydrogenase (LDH > 2.5 upper limit of normal) or patients considered at a high risk of thrombosis in the opinion of the investigator.

### Baseline assessment

All patients had a detailed baseline assessment including clinical review, laboratory blood tests, blood samples for NAb and ELISPOT, resting transthoracic echocardiography with LVAD at standard settings and a reduced flow setting, cardiopulmonary exercise test and 6-min walk test.

### Randomisation

Following baseline assessment and NAb testing, patients were randomised in a 2:1 ratio to receive either an intracoronary infusion of 1 × 10^13^ DNase resistant particles AAV1/SERCA2a or placebo (buffer solution without viral particles), stratified in a 1:1 ratio by NAb status (positive (≥1:2) or negative). Randomisation was performed using an interactive web-based randomisation system linked to the InForm Trial Management system. Randomisation codes were generated by an independent statistician using blocked randomisation.

### SERCA2a gene therapy administration

The intracoronary infusion protocol was performed using the same technique applied in the CUPID2 trial [16]. Briefly, patients were admitted and oral anticoagulation interrupted using a patient-specific bridging anticoagulation strategy (either intravenous unfractionated heparin or subcutaneous low molecular weight heparin). Vascular access was obtained via the femoral artery using ultrasound guidance and coronary artery anatomy was confirmed with diagnostic coronary angiography. Femoral artery access was chosen to allow real time mean arterial blood pressure (MAP) monitoring during the GTN infusion and IMP delivery. Intravenous GTN infusion was initiated 25 min prior to infusion of the investigational product as per the CUPID 2 trial protocol, starting at 5 mcg/min and increasing in 5 mcg/min increments every 3–5 min until a criterion was met that defined maximum tolerated dose. These criteria were (i) LVAD flow dropping by >0.5 litres/min, (ii) MAP falling below 65 mmHg, or (iii) MAP falling >10 mmHg from baseline. Once the maximum tolerated dose was reached, this was continued until the end of the IMP infusion. All patients had continuous flow LVADs and the median MAP was 79 mmHg (76–84 mmHg (IQR)). Heparin was delivered systemically to achieve a target activated coagulation time (ACT) of 200 s prior to starting the intracoronary infusion.

The IMP was prepared according to trial pharmacy protocol and delivered as a 50 ml intracoronary infusion to the left ± right coronary arteries at 5 ml/min according to the coronary anatomy to maximise delivery to the LV myocardium.

After the infusion, the ACT was rechecked. The strategy for managing the arterial access site was decided on a case by case basis, guided by the location of femoral puncture and ACT at the end of the procedure. Options included deployment of a vascular closure device, immediate removal and manual pressure, or delayed removal and manual pressure. Anticoagulation with IV heparin and oral anticoagulation was then restarted.

### Safety endpoints

Patients were reviewed on a weekly basis for the first 4 weeks following IMP infusion, and then at monthly intervals for months 2–6. Clinical assessment, review for adverse events, laboratory blood tests including cardiac troponin, liver transaminases, C reactive protein (CRP) and creatine kinase (CK) were performed at each visit. ELISPOT testing was performed at baseline, M3 and M6, and if clinically indicated, e.g., unexplained fever (see detailed ELISPOT methods in data supplementary).

### Functional endpoints

Exploratory efficacy studies were performed at baseline, M3 and M6 (pre biopsy). Resting transthoracic echocardiography was performed with LVAD at standard and reduced impeller rotation speed (see detailed Methods and Tables S2–4 in data supplementary). Exercise capacity was assessed by cardiopulmonary exercise testing (CPEX) using the modified Bruce protocol and by six minute walk testing (6MWT), both with LVAD at normal speed and reduced speed (see detailed methods in data supplementary). Blood was collected for B-type natriuretic peptide (BNP) measurement.

### Left ventricular endomyocardial biopsies

Left ventricular EMBs were taken at 6 months post gene transfer unless a patient had undergone cardiac transplantation during the 6 month period post IMP infusion. Oral anticoagulation was interrupted with bridging using the same protocol as described above for the IMP infusion. The LV biopsy was performed using a percutaneous bioptome via the femoral artery under fluoroscopic guidance. After crossing the aortic valve, 3-6 samples were taken from the LV endocardial surface. Samples were visually inspected to ensure myocardial tissue was present, and if fibrotic tissue was observed further samples could be taken. Biopsies were immediately frozen in liquid nitrogen and stored at −80 °C until analysis.

### Detection of transgene

The presence and copy number of the AAV1/SERCA2a DNA were measured by quantitative PCR, and the expression levels of human endogenous and vector-derived SERCA2a mRNA in human LV samples by RT-qPCR (see data supplementary). Results are expressed as copy number of single-stranded AAV1/SERCA2a vector per microgram (µg) of patient tissue DNA.

### Laboratory cell and tissue studies following cardiac transplantation

Explanted hearts were transported to the laboratory in cardioplegic solution within 6 h of removal. Cardiomyocytes were isolated for contraction, electrophysiology and morphological studies. Human myocardial tissue slices were prepared and studied using multielectrode arrays. For detailed methods see data supplementary.

### Statistical analysis

The primary outcome of the trial was to understand the safety and feasibility of administering the SERCA2a gene via an AAV1 viral vector in humans with HF. This was to be evaluated based on incidences of death, major adverse cardiovascular events and out-of-range laboratory values across 24 patients randomised at a 2:1 ratio between AAV1/SERCA2a and placebo and stratified by the presence of NAbs.

The trial was terminated early with only 1 subject allocated to the placebo arm and only 1 subject within the positive antibody stratum. Applying statistical testing such as Students *t* test would not be appropriate. Therefore the trial results are presented as descriptive data from the five randomised subjects. Statistical tests for the cell and tissue data are described in the Figure legends.

## Results

**Fig. 1:**
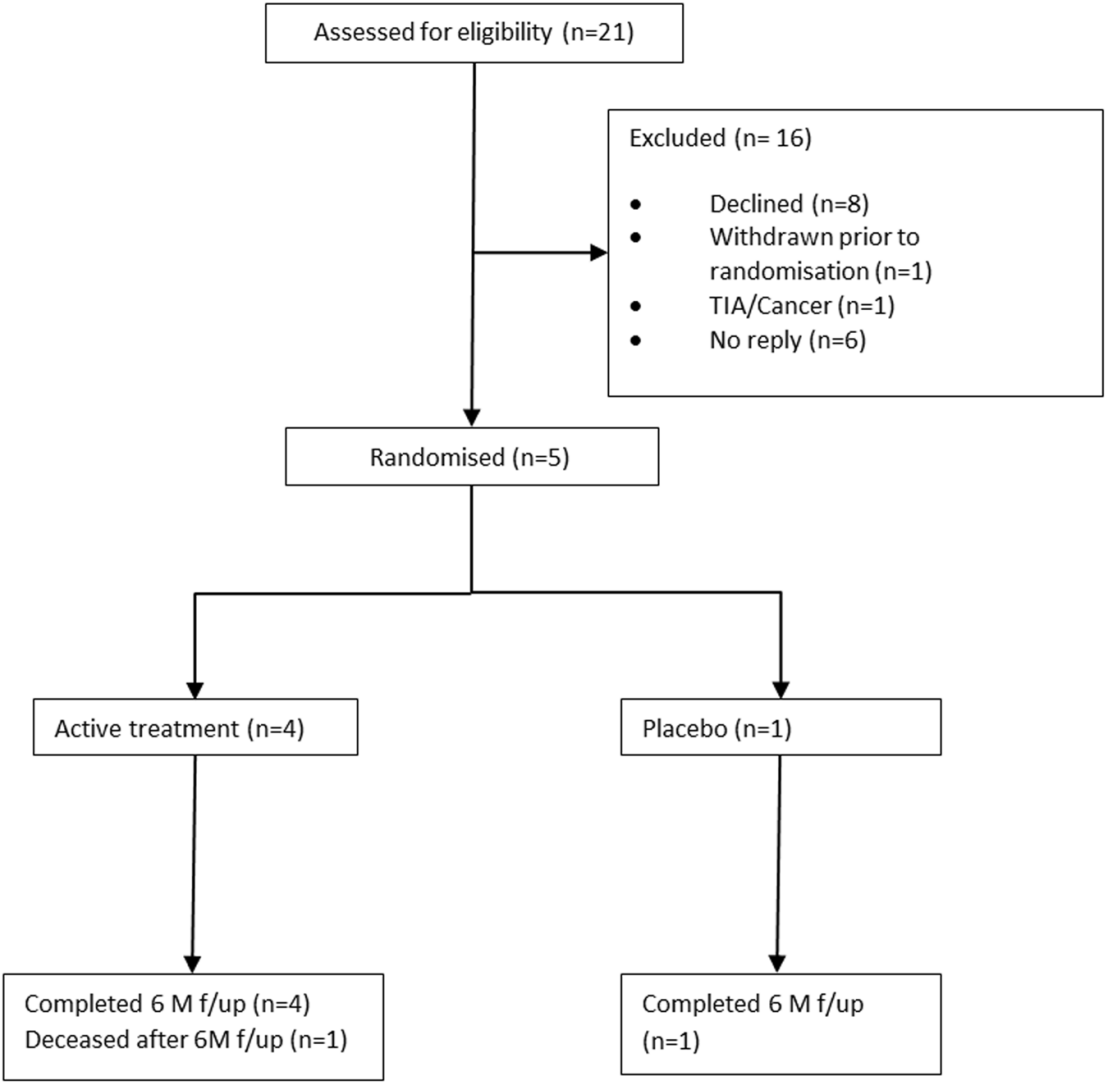
Trial profile: Consolidated Standards of Reporting Trials (CONSORT) diagram.

Recruitment started at Harefield Hospital in July 2014 and was stopped after the TSC reviewed the provisional results of the CUPID 2 trial in May 2015. Screening the population of adults with LVADs for chronic HF identified 21 individuals who were approached to discuss the study. At the time of stopping trial recruitment six subjects had provided informed consent, one of whom subsequently withdrew prior to IMP administration (see Fig. 1). Papworth Hospital had not been activated at the time recruitment was halted. Five subjects were randomised and received IMP infusion. Four received AAV1/SERCA2a gene therapy infusion (trial subjects 1–3 and 5) and one received placebo infusion (trial subject 4). Baseline characteristics are presented in Table 1. Four patients (trial subjects 2–5) had non-ischaemic dilated cardiomyopathy and one patient (1) had chronic HF secondary to severe aortic valve stenosis which failed to recover following bioprosthetic aortic valve replacement (AVR). All five patients were treated with warfarin and aspirin for prophylaxis against LVAD-associated thrombosis, and all received guideline-based HF pharmacological treatment.

**Table 1:** Baseline characteristics.

	Subject				
	001	002	003	004	005
Treatment	AAV1/SERCA2a	AAV1/SERCA2a	AAV1/SERCA2a	Placebo	AAV1/SERCA2a
Strata	AAV −ve	AAV +ve	AAV −ve	AAV −ve	AAV −ve
Age (Years)	36	69	29	49	30
Sex	M	M	M	M	F
Ethnicity	White	White	White	Black	White
BMI	21.45	23.20	27.07	34.25	23.46
Cause of Heart Failure	Valvular Heart Disease	Dilated cardiomyopathy	Familial cardiomyopathy	Dilated cardiomyopathy	Familial cardiomyopathy
Type of LVAD	Heartware HVAD	Heartware HVAD	Thoratec Heartmate 2	Heartware HVAD	Heartware HVAD
Duration of Optimal HF Regime (months)	1	19	3	27	14
Cardiac Transplant Waiting List?	Yes	Yes	Yes	Yes	Yes
Creatinine (umol/L)	62	81	130	128	70
6MWT (metres)	623	627	480	563	397
Peak VO_2_ (ml/kg/min)	28.30	19.60	20.30	13.50	15.60

Four patients (1, 3–5) were NAb negative and one (2) was NAb positive (assay result ≥ 1:16) (see Table 2). This subject (2) received AAV1/SERCA2a gene therapy, and to our knowledge this is the first patient recruited into a cardiac gene therapy trial for HF using AAV vector who was NAb positive. At 6 months repeat NAb testing confirmed subjects 1 and 3 had seroconverted consistent with exposure to AAV1/SERCA2a. This suggests that the virus had been delivered correctly and was active and removes one possible explanation for the lack of gene transfer. Subject 2 remained positive, and subject 4 who received placebo and was NAb negative at baseline remained NAb negative at 6 months. No 6 month data was available for subject 5 (see below).

**Table 2:** Neutralizing antibody status at baseline and 6 months.

Subject	Treatment	Baseline NAb	Baseline AAV1 neutralizing antibody titre	M6 AAV1 neutralizing antibody titre
1	AAV1/SERCA2a	AAV −ve	<1:2	>1:64
2	AAV1/SERCA2a	AAV +ve	>1:16	>1:64
3	AAV1/SERCA2a	AAV −ve	<1:2	>1:64
4	Placebo	AAV −ve	<1:2	<1:2
5	AAV1/SERCA2a	AAV −ve	<1:2	–

### Safety results

There were four serious adverse events (SAEs) reported during the trial active phase and none were judged to be related to AAV1/SERCA2a.

**Fig. 2:**
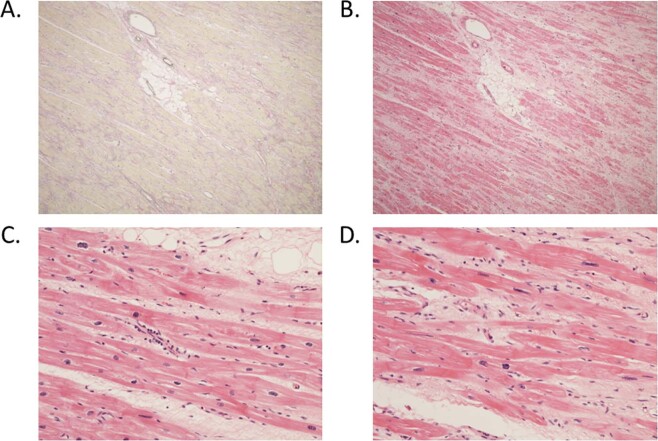
Histopathology from human hearts following AAV1/SERCA2a gene delivery. Both hearts showed similar features with the myocardium showing patchy fine interstitial fibrosis around myocytes with focal areas of more confluent fibrous replacement (Fig. 2a Elastin Van Gieson (x40) and **b**. Haematoxylin &Eosin (x40)). There was no evidence of endocarditis or myocarditis in either specimen and, except for rare perivascular lymphocytes seen focally in one specimen (Fig. 2c), no inflammatory infiltrates were seen in either heart. This was associated with some myocyte hypertrophy with nuclear enlargement (Fig. 2d). The features in both specimens were consistent with dilated cardiomyopathy without evidence of inflammatory myocarditis.

Three SAEs were related to subject 5 who received AAV1/SERCA2a. This subject was admitted 74 days after IMP infusion for right HF decompensation requiring inotrope support and leading to urgent transplant listing. This was a clinical deterioration due to underlying disease progression in the opinion of the investigators. Subject 5 had been noted to have an elevated troponin at baseline before AAV1/SERCA2a infusion, and there was no further rise in troponin after IMP infusion with CK, WCC and CRP remaining normal. Subject 5 had no clinical features to support the diagnosis of myocarditis clinically and their ELISPOT at M3 was negative. Subject 5 proceeded to urgent cardiac transplantation on day 165 following IMP infusion. Their explanted heart was collected for clinical assessment and research samples for PCR. There was no evidence of inflammatory infiltrate on histopathological assessment (see Fig. 2). Unfortunately the post-operative course was complicated by recurrent sepsis and multiorgan failure, and subject 5 died 4 weeks post cardiac transplantation and 28 weeks post IMP infusion. The clinical course and death was reviewed by the local principal investigator and the data safety monitoring committee and not judged to be IMP related.

The fourth SAE occurred in subject 3 who also received AAV1/SERCA2a. This subject was hospitalised for assessment of LVAD alarm 51 days following IMP infusion. Blood tests and clinical assessment were normal, with no evidence of LVAD thrombosis or haemolysis, and the subject was discharged home after a period of observation without any change to treatment.

There was one procedure-related vascular complication with a haematoma related to the femoral artery access site (subject 1 – AAV1/SERCA2a), and one subject (4 – placebo) developed a new atrial flutter 26 days following their infusion. There were no other AEs potentially attributable to the infusion or EMB procedures.

Following the active trial phase subject 3 (receiving AAV1/SERCA2a) also proceeded to cardiac transplantation following a chronic deterioration at 1 year 10 months after IMP infusion.

There were no clinically significant changes in haemoglobin, renal function or liver enzymes on safety blood tests. One subject (4 – placebo) had a new troponin rise 1 week following IMP infusion (see Table S5). The troponin remained elevated from week 1 to month 5, and was normal at baseline and 6 months. The subject was clinically stable with no new symptoms and specifically no symptoms or signs suggestive of a systemic inflammatory reaction or cardiac complication. Other blood tests including white cell profile, CK, LDH, CRP and liver enzymes remained normal. An additional ELISPOT sample was taken which was normal. This subject received placebo and therefore the troponin rise was not related to AAV1/SERCA2a, but could possibly have been related to the study infusion procedure given the temporal correlation. Subject 5 had an elevated troponin at baseline and it remained stably elevated without a significant change. Subject 3 had a single borderline measurement (40 ng/L) at M3 without any clinical or biochemical abnormalities.

In summary, none of the patients receiving AAV1/SERCA2a had an AE attributable to AAV1/SERCA2a, but there was one definite (haematoma) and two possible (asymptomatic troponin rise, atrial flutter) AEs related to the infusion procedure. There was no evidence for viral-induced immunoreactions or myocarditis in this small cohort.

### Human myocardial gene transfer

Percutaneous EMB were scheduled for subjects 1–3 at 6 months post IMP infusion as per trial protocol. Only one of the three procedures yielded myocardial tissue suitable for PCR analysis (subject 3). Viral transgene DNA was detectable at low copy number (38 ssDNA copy number/µg human DNA) in one of the four EMB samples, originating from the inferobasal LV wall (see Table 3). Multiple EMB samples from subject 2 were fibrotic macroscopically with no samples suitable for PCR. It was not possible to cross the bioprosthetic AVR of subject 1 despite reducing LVAD flow rates and no EMB samples were taken. Following the results of CUPID 2 trial and the decision to halt recruitment, the decision was also taken not to pursue EMB in the remaining two subjects given the potential risks involved.

**Table 3:** AAV1/SERCA2a vector DNA presence in the heart by qPCR.

Subj.	Treatment	AAV NaB Strata	Source	Sample	AAV1/SERCA2a DNA Conc.^a^
3	AAV1/SERCA2a	−ve	Basal Interior Wall	EM Biopsy	38.0
			Basal Interior Wall	EM Biopsy	BLOD
			Lateral Wall	EM Biopsy	BLOD
			Lateral Wall	EM Biopsy	BLOD
3	AAV1/SERCA2a	−ve	Anterior Septum	Transplant	BLOD
			Anterior Wall	Transplant	BLOD
			Posterior Septum	Transplant	BLOD
			Posterior Wall	Transplant	BLOD
5	AAV1/SERCA2a	−ve	Anterior Septum	Transplant	80.0
			Anterior Wall	Transplant	41.1
			Posterior Septum	Transplant	56.7
			Posterior Wall	Transplant	23.1

*BLOD* below limit of detection.

^a^ss DNA copy numbers/µg human gDNA.

Subjects 3 and 5 proceeded to cardiac transplantation. Twelve samples of LV myocardium from the anterior, septal and posterior walls were studied. Viral transgene DNA was detected at a low level in tissue from Subject 5 (anterior septum 80.0 ssDNA copy number/µg human DNA; anterior wall 41.1; posterior septum 56.7; posterior wall 23.1, mean of 3 samples each) but not detectable in the LV samples of subject 3 at 22 months post AAV1/SERCA2a infusion, despite one of this patient’s EMB having been positive at 6 months (see Table 3).

### ELISPOT results

ELISPOT measurement allowed serial assessment of immunoreactivity to AAV in patients in the trial. Results were available for subjects 2–5. The specific AAV1 ELISPOT was negative in all subjects at baseline, and became positive in two subjects (2, 3) at 3 months, with persisting positive ELISPOT result in subject 2 (NAb positive subject) at 6 months and remained negative in the other subject who received AAV1/SERCA2a virus. There was no correlation with any clinical or laboratory changes to suggest a clinical immunoreaction, and trial investigators were blinded to ELISPOT results until the end of the trial. The positive ELISPOT and its persistence are interesting as ELISPOT positivity was not observed in CUPID and other trials at a dose of 10^13^ vg.

### Histopathology

Two patients proceeded to cardiac transplantation (subjects 3 and 5), both of whom were NAb negative and received AAV1/SERCA. No inflammatory infiltrates were seen in either heart, whereas patchy fine interstitial fibrosis around myocytes, both individually and in groups, with focal areas of more confluent fibrous replacement and myocyte hypertrophy with nuclear enlargement was observed, consistent with dilated cardiomyopathy (see Fig. 2).

### Exploratory efficacy outcomes

Functional endpoints were assessed by exercise testing (CPEX and 6MWT), resting echocardiography at normal and low LVAD speed, and BNP.

CPEX and 6MWT proceeded as per protocol except for subject 5 at M3 and M6, when the patient’s clinical status curtailed full assessment, and for subject 2 at M6 when the CPEX data were incomplete. Fourteen echocardiograms were performed on the 5 subjects. The fifteenth scheduled echo scan was not performed as the patient was critically ill awaiting cardiac transplantation (subject 5, M6). These 14 scans included 10 low speed assessments, and in each of these the target low speed was reached (HeartMate II, 6,000 rpm; HVAD, 1800 rpm; see Supplementary Method) and there were no complications. Four low speed assessments were omitted, three of these due to suspension of the trial. The fourth (in subject 3 at M3) was omitted due to a LVAD alarm and possible haemolysis episode occurring in the interval between 0 and M3 assessments (described above), and the theoretical risk of exacerbating or precipitating an LVAD thrombosis. Data obtained from echocardiography was limited by quality of ultrasound windows, constrained by apical placement of the LVAD.

There were no consistent changes in any of peak oxygen consumption (peak VO_2_), change in ventilatory equivalent for carbon dioxide slope, 6 min walk distance, LV end diastolic dimension at normal LVAD speed, or BNP, across the whole cohort or stratified by treatment group (see data Supplementary Table S6). In subjects for whom data was available, there were no consistent changes in LV ejection fraction or LV global circumferential strain at normal or low LVAD speed.

There was a pronounced increase in BNP at M3 (359–477 ng/L, 33% increase) in subject 5 consistent with that subject’s clinical deterioration. This had decreased to 344 ng/L at 6 months, but this probably reflects rescue inotropic therapy rather than improvements in native heart function. There was no significant change in other patients. Full results are included in the data Supplementary Table S6.

### Laboratory cellular and tissue studies (see supplementary)

Tissue was obtained from the explanted hearts of the two subjects (3 and 5) who had undergone heart transplantation: both were from the group treated with AAV1/SERCA2a and NAb negative. This was compared with control experiments on contemporaneously collected myocardial tissue from a donor hearts unsuitable for transplantation after explanation (*n* = 2), and from HF patients with DCM (*n* = 8), ICM (*n* = 3), myocarditis (*n* = 1) and HCM (*n* = 1) whose hearts were explanted for transplantation. Data were collected from isolated ventricular cardiomyocytes and ultrathin myocardial slices, while tissue blocks were analysed by electron microscopy (EM) and electron tomography. These are described in detail in the Supplementary (see Figs. S1–3).

## Discussion

This is the first clinical trial of gene therapy in patients with LVADs for chronic HF. The SERCA-LVAD trial did not complete recruitment due to the results of CUPID 2 which became available during the study and altered the risk:benefit ratio for this patient cohort leading to cessation of recruitment and inability to obtain planned EMB for 2 subjects. This is a major limitation of the trial, and interpretation and discussion of the results reflects the observational nature with the lack of power for any statistical analyses.

The primary aims were safety and feasibility. There was no suggestion of a safety concern in this small cohort, with none of the four subjects undergoing gene therapy displaying any clinical or laboratory abnormalities directly attributed to administration of the AAV1/SERCA2a vector at the dose studied. One patient deteriorated 10 weeks post administration, and required hospitalisation for HF, inotropic support and proceeded to cardiac transplantation. The local PI and trial steering committee concluded this deterioration reflected the natural history of the underlying disease in this subject, and there was no objective evidence of a viral mediated immune reaction or myocarditis as a contributory factor. ELISPOT, CK, WCC and CRP were all normal in the subject at M3 post infusion, and cardiac troponin which was raised at baseline, reflecting advanced HF, did not significantly rise from baseline. Histopathology also excluded an inflammatory infiltrate (Fig. 2). However, given the limited experience of AAV gene therapy in patients with HF, and specifically in patients with LVADs, absolute exclusion of a contributory effect of the gene therapy infusion in the subsequent clinical deterioration of subject 5 is not possible, although the investigators’ view is that there is no evidence of a causal relation. One procedure related AE (femoral artery access site haematoma) should be noted, which reflects the complexity of performing angiography in LVAD patients with reduced pulse pressure and complex anticoagulation requirements and the need for real time arterial BP monitoring during IV GTN infusions.

Five trial subjects with LVADs received IMP infusion, with four receiving AAV1/SERCA2a gene therapy. This trial confirmed the feasibility to perform this procedure in this patient group, and in one case EMB at 6 months confirmed the presence of viral transgene DNA. Analysis of myocardial samples from one of the two transplanted hearts confirmed detection of exogenous AAV1/SERCA2a DNA confirming delivery to the human failing heart (Table 3). It was also feasible to perform a range of cellular and tissue research studies in explanted human hearts following gene therapy (Figs. S1–S3).

There are several challenges that need to be overcome if future gene therapy trials with larger patient cohorts are to be successful. Firstly, the patient cohort with LVADs is limited in number compared with the wider chronic HF population. The LVAD patient cohort are a younger population compared with the general HF population, but have an important unmet clinical need of long term outcome and weaning strategies, and our experience was that this patient cohort is engaged to participate in research studies of advanced medicinal products such as gene and stem cell therapies. The unselected LVAD population have many co-morbidities, and with LVAD-associated thrombosis an ongoing concern, there are a number of factors limiting the population suitable for research trials where anticoagulation needs to be interrupted for invasive procedures. These factors raise the challenge of the absolute population of LVAD patients eligible to participate in a trial of this type, and the number of centres required.

Secondly the readout of efficacy is more challenging in HF patients with LVADs compared with those without. Clinical parameters such as LVEF and natriuretic peptides can be measured but are greatly influenced by the impact of the associated LVAD, introducing significant variability which requires large cohort numbers to overcome. For gene therapy trials the ability to measure exogenously delivered DNA via PCR is an attractive strategy as proof of delivery. However, the requirement for myocardial tissue presents a challenge in a patient cohort where stable LVAD patients may undergo prolonged LVAD support without proceeding to transplantation, and in several countries a growing proportion of the LVAD population have destination therapy and are ineligible for transplantation. To overcome these limitations, we introduced EMB into the trial protocol for participants who had not proceeded to cardiac transplantation in the 6 months following IMP infusion. Whilst LV EMBs are possible in this patient population, they raise a number of important scientific and safety issues. The myocardium of patients with advanced HF who require LVAD support may have significant amounts of fibrosis due to the underlying disease, particularly but not exclusively if they have coronary disease and prior myocardial infarction. Following long term LVAD support endocardial fibrosis may develop, limiting the quality of EMB samples suitable for PCR analysis. In the two subjects where EMB was technically possible and performed, one had multiple samples taken but all were too fibrotic for analysis. EMB in this patient cohort is technically challenging, both for safety (avoiding the LVAD inflow cannula in the LV, risk of cardiac rupture in a thinned myocardium), and practical reasons (crossing the aortic valve in a retrograde direction can be challenging if aortic valve opening is reduced on LVAD support). Peri-procedural management of anticoagulation is complex, and interrupting stable anticoagulation for a research procedure is an important factor to consider in the context of LVAD-associated thrombosis with continuous flow devices.

Human myocardium was obtained from three trial subjects via EMB or cardiac transplantation, although it was only suitable for myocardial analysis from two subjects, as the EMB from subject 2 were too fibrotic. Viral transgene DNA was detectable using PCR in one biopsy and one transplant sample, but at low copy number, and absent in another biopsy and transplant sample (Table 3). These low or absent levels are consistent with the previously reported levels of transgene DNA measured in patients enroled in CUPID 1 and CUPID 2 trials who proceeded to LVAD implantation or cardiac transplantation (reported levels in range <10–192 ssDNA copy number/µg human DNA in samples from 8 patients in CUPID 2: see Table S7) [11, 12]. In AGENT-HF, myocardium was obtained from one patient at the time of explant at 18 months, but again no AAV1/SERCA2a DNA was detected by qPCR [13]. In the CUPID 1, CUPID 2 and AGENT-HF trials there is the potential bias that LV myocardium was only available from patients that clinically deteriorated, and therefore by definition had not received a potential therapeutic benefit from the trial intervention. In this current trial, the prospective planned collection of tissue from all trial subjects was designed to overcome that bias. However in the context of the CUPID 2 results where no clinical benefit was observed in the active gene therapy arm across a range of clinical parameters, the result from this trial with low or absent levels of transgene DNA, would support the hypothesis that the main reason for lack of benefit observed with AAV1/SERCA2a gene therapy at the dose studied in CUPID 2, AGENT-HF and SERCA-LVAD trials was the failure to deliver the transgene at sufficiently high levels to the failing human heart to have an impact upon the underlying disease pathophysiology. The low or absent levels of viral transgene DNA in this trial and the CUPID 2 trial is in contrast to much higher levels in preclinical small and large animal models of HF where SERCA2a was tested (range 8000–42,000 ssDNA copy number/µg DNA). The potential reasons for this failure of gene delivery and clinical impact are multiple, including the dose studied being too low, high first pass washout in the coronary circulation, lack of proof of functional protein expression and elevated SERCA2a enzymatic activity with the associated changes in cardiomyocyte calcium physiology observed in preclinical models. Other gene therapy trials for the treatment of monogenic diseases have used much higher doses of AAV vectors [17–20] however there are limits to how much the doses can be increased before toxicity occurs [21].

One new aspect of this trial was the recruitment of patients with NAbs to the viral vector to test the hypothesis of whether they prevented transfection efficiency and also if there are any safety concerns. This is the first cardiac gene therapy trial to enrol NAb positive patients, although subjects recruited in other gene therapy trials have been reported. Only one of our 5 subjects was NAb positive (Table 2), in contrast to the much higher rates (59%) of detectable NAbs in the general HFrEF population prescreened for the CUPID 2 trial and our general HFrEF population, which may reflect the younger cohort in comparison with the CUPID trials. There were no safety concerns in our trial subject who was NAb positive and received AAV1/SERCA2a gene therapy, but it was this subject who had positive ELISPOT results on blood tests taken at 3 and 6 months which may reflect amplification of the T cell response due to previous exposure. Unfortunately, the EMB in this patient did not yield tissue for analysis, but given that we only had a single patient in this group, and also that gene transfer was low or absent in other subjects, it is unlikely to have provided an answer at the dose studied. Definitive data regarding safety and whether efficacy of delivery is affected by NAb requires further studies with larger number and at higher AAV doses.

In summary, the SERCA-LVAD trial is the first trial of gene therapy for patients with HF and LVADs. It was feasible to deliver the AAV1/SERCA2a gene therapy infusion in this patient cohort, and DNA was detectable at follow up EMB in one case and one explanted heart. The detected level of DNA was very low, and absent in the other second explanted heart analysed which had the positive EMB result, supporting the observation from the CUPID 2 and AGENT-HF trials that the gene therapy was not effectively delivered and the dose and protocol applied in these trials, and this may explain the neutral result of CUPID 2 trial on clinical outcomes [12, 13].

The main impact of the SERCA-LVAD trial may be to alert the Advanced Therapies field to the challenges of using this patient cohort. It is frequently suggested that LVAD patients might have advantages, for example the possibility to add vectors (or regenerative patches) and sample myocardium in an open-chest procedure as the LVAD is implanted. However, the reality is that the instability of the clinical condition during and immediately after LVAD implantation precludes reliable baseline measurements of cardiac function or blood and tissue markers. In addition, we have now observed the complications of intra-arterial delivery of a vector when there is no pulse; the issues regarding anticoagulation management during vector delivery or biopsy; the difficulty of measuring cardiac function with the LVAD supporting LV function; and the dilemma of whether to immunosuppress for a positive ELISPOT when the LVAD patient is highly susceptible to drive-line infections. Overall, these safety concerns are significant, especially for untried Advanced Therapies where potential benefit is far from defined, and the SERCA-LVAD trial is the first to identify and address these challenges and aid the design of future trials in this field.

## Supplementary information

Supplementory methods and results

Figure S1

Figure S2

Figure S3
