# Supplementary material for: Investigation of the safety and feasibility of AAV1/SERCA2a gene transfer in patients with chronic heart failure supported with a left ventricular assist device – the SERCA-LVAD TRIAL
Source: Gene Ther. 2020 Jul 15;27(12):579–90. doi: 10.1038/s41434-020-0171-7 (PMC7744277; doi:10.1038/s41434-020-0171-7)
Supplement: Supplementary file 1 — Supplementory methods and results [file 41434_2020_171_MOESM1_ESM.docx]

## Methods

### Trial recruitment and eligibility criteria

Table S1. Inclusion and Exclusion Criteria

| Inclusion criteria | 1. Patients that have had a left ventricular assist device (LVAD) implanted for chronic heart failure, where chronic heart failure is defined as at least 6 months 2. Patients are clinically stable in the opinion of the clinical team looking after the patient 3. Written informed consent |
| --- | --- |
| Exclusion criteria | 1. <18 or >70 years of age at the time of consent 2. Pregnancy or within 6 months of giving birth 3. Women of child-bearing potential not using an effective method of contraception 4. Men not using an effective method of contraception 5. Suspected or active bacterial, viral, fungal or parasitic infection within 48 hours prior to administration of IMP, in the opinion of the investigator*. 6. Patients at a high risk of thrombosis in the opinion of the investigator 7. Patients with a previous episode of LVAD thrombosis on their current device 8. Patients with persistently raised lactate dehydrogenase (LDH >2.5 ULN) 9. Patients requiring triple anticoagulation i.e. warfarin and dual anti-platelet 10. Patients participating in another clinical trial |

* Eligible, enrolled and randomised patients who develop an infection will have study treatment delayed until 7 or more days after the time point when infection is no longer clinically evident.

ULN Upper limit of normal range

### Echocardiography and Cardiopulmonary Exercise testing (CPEX)

### **i. Echocardiography protocol including low speed assessment**

The transthoracic echocardiography (TTE) protocol was conceived to allow structural and functional cardiac evaluation, and to identify response to study intervention. The TTE was performed with the LVAD at normal clinical operating speed, and with the LVAD speed turned down to the minimal safe and/or tolerated level.

All studies were performed using the Phillips IE33 at Harefield Hospital. This machine was serviced and maintained to clinical standard according to institutional protocols. For all the low speed assessments there were three researchers present: in addition to the clinical research fellow (coordinating the study and collecting the data) there was a consultant cardiologist performing the echocardiogram and an experienced VAD nurse managing the patient, VAD equipment and monitoring clinical status.

On arrival the patient’s study consent was checked and new verbal consent was obtained, reiterating the small risk of thromboembolism and pump thrombosis. Blood pressure and LVAD speed/power were recorded at baseline. Continuous ECG monitoring was used during the study. A core dataset of parameters was obtained with the LVAD running at normal clinical operating speed (see Table S2).

Table S2: Echocardiographic parameters obtained during baseline study

| **Ventricular function assessment:**   - PLAX M-mode dimensions - Biplane LV end diastolic and end systolic volumes, and LV ejection fraction, by Simpson’s method - Three loops of 2D speckle with highest frame rate technically possible in PSAX, A4C and A2C, for post hoc strain assessment. - Assessment of RV function using TAPSE   **Valve assessment:**   - Colour flow looking for aortic regurgitation (AR) including AR jet as % LVOT diameter and AR vena contracta - Visual assessment of mitral regurgitation and where possible assessment using the PISA method - Visual assessment of tricuspid regurgitation and measurement of TR Vmax   **VAD assessment**   - Right and left high parasternal recordings (with Doppler where possible) to visualise VAD outflow |
| --- |

The low speed element of the study was completed immediately following the initial scan. To minimise risk of pump thrombosis this was done as quickly as possible, with a maximum time limit of 45 minutes. This allows 15 minutes for ramp down, 10 minutes wait time, 5 minutes acquiring data and 15 minutes for ramp up to normal speed.

Prior to any speed adjustment the anticoagulation regime was reviewed and action taken as shown in Table S3. If the INR was outside the patient’s target range, the patient’s warfarin dosing was reviewed post-procedure.

Table S3. Anticoagulation management

| ***INR on day of procedure*** | ***Anticoagulation management*** |
| --- | --- |
| ***<1.5*** | Postpone procedure until INR>2 |
| ***1.5-1.9*** | Give 10,000 unit heparin bolus before starting |
| ***2.0-2.9*** | Give 5,000 unit heparin bolus before starting |
| ***3.0-4.0*** | Proceed without heparin bolus |
| ***> 4*** | Postpone procedure until INR<4 |

Ramp down: The LVAD speed was reduced in increments every 1 minute with BP and echo assessment at each stage. The aim was to reduce the speed to the minimum safe speed or as far as tolerated by the individual patient. The typical clinical operating speeds, size of incremental reduction and minimum safe speed vary between devices and are shown in Table S4. The speed was never reduced below the minimum speed specified, and the total time below the patient’s clinical operating speed never exceeded 45 minutes.

Table S4. Clinical operating speeds and incremental reductions used in the low speed protocol

| (rpm = revolutions per minute) | ***Thoratec***  ***HeartMate II*** | ***Heartware***  ***HVAD*** |
| --- | --- | --- |
| ***Typical clinical operating speeds*** | 8,000-9,000rpm | 2,500-3,200rpm |
| ***Reduce in increments of*** | 200rpm | 100rpm |
| ***To minimum speed*** | 6,000rpm | 1,800rpm |

Once at minimum speed or lowest speed tolerated there was a 10 minute wait time for equilibration. At the end of the wait time, BP was recorded and LV studies were repeated (‘Ventricular function assessment’ in Table S2).

As soon as the echo studies were complete, the LVAD was returned to baseline speed, using incremental steps at 1 minute intervals using echo and BP monitoring, and by the same rpm increments used for speed reduction. At the end of the study, the pump power consumption was recorded. Thereafter the patient was instructed to check the pump power every 15 minutes for 2 hours post procedure, as a sustained increase could suggest pump thrombosis. If patient or researcher were concerned then the patient returned the following day for blood examination for haemolysis markers.

## ii. Cardiopulmonary exercise testing protocol

Cardiopulmonary exercise (CPEX) testing forms a key part of functional clinical assessment in HF and particularly in cardiac transplantation and MCS. All the CPEX testing was done with the LVAD running at full clinical speed.

All CPEX testing was done with the exercise physiologist, VAD nurse and clinical research fellow present. All testing was done at Harefield Hospital using the Ultima CardiO2 gas exchange analyser. This and the treadmill were serviced and maintained to clinical standard according to institutional protocols, including calibrated for flow and gas analysis done daily as per manufacturer instructions. Other required equipment was a Doppler probe for BP recordings, and card for demonstrating the Borg scale. If the patient was fitted with an intracardiac defibrillator there was a pacing physiologist in attendance.

On arrival the patient’s study consent was checked and new verbal consent was obtained. Blood pressure (using Doppler probe if necessary) and LVAD speed/power were recorded at baseline. Continuous ECG monitoring was used during the study. A minimum 5 minutes resting gas exchange data was obtained prior to starting exercise^[[1]](#footnote-1)^. If the patient had an ICD, then for patient safety, the VT therapies were turned off, and VF detection threshold to >200bpm^[[2]](#footnote-2)^.

Exercise was commenced as per the modified Bruce protocol. The Borg dyspnoea score was obtained every minute during the test. Blood pressure (BP) measurements were not attempted during the test as clinical experience states these are unreliable during exercise. Exercise was discontinued immediately if adverse events occurred. BP measurement was obtained using the Doppler probe immediately after exercise, and the patient was monitored for 5 minutes after completion. In patients with an ICD, therapies were reprogrammed to pre-test settings before leaving the exercise room.

The following data parameters were recorded for each CPEX test: duration of exercise (minutes); final Borg score before cessation of test; stage of exercise protocol reached; peak oxygen consumption (VO_2_; mls/kg/min); VE/VCO_2_ slope (average of whole test); respiratory exchange ratio; reason for stopping.

### PCR analysis of AAV1huSERCA2a vector and SERCA2a mRNA in human left ventricular samples

The presence and copy number of the AAV1/SERCA2a DNA by quantitative PCR, and the expression levels of human endogenous and vector-derived SERCA2a mRNA in human left ventricular samples by RT-qPCR. Snap frozen 0.5cm^3^ portions of the left ventricular tissue from anterior and posterior septum as well as from anterior and posterior wall were weighed and stored at -80°C. For purification of total DNA and RNA, tissues were lysed individually to avoid cross contamination between samples. Genomic DNA was isolated by using QIAamp DNA Mini Kit (Qiagen). The concentration and quality of gDNA was analysed by NanoDrop 8000 spectrophotometer. A260/280 ratio of DNA samples were ≥1.9 in each sample. The potential presence of AAV1/huSERCA2 in samples (125 ng is used per reaction) was analysed by qPCR; each sample was measured in triplicate wells. Total RNA from heart tissues was also isolated from the lysates using a Qiagen RNeasy Mini Kit and quantitated by NanoDrop 8000. 700 ng of RNA samples were reverse transcribed into cDNA by High-Capacity cDNA Reverse Transcription kit (Thermo Scientific). Expressions of human endogenous and vector-derived SERCA2a transcripts were measured by TaqMan qPCR assays using 2x TaqMan Universal PCR Master Mix (Thermo Scientific) and the following primers/probe specific for vector-derived SERCA2a, human endogenous SERCA2a, and human GAPDH as housekeeping gene, respectively. The DNA primers and probe which amplify a 107-nucleotide sequence from AAV1/SERCA2a cDNA (3' end of SERCA2 and junction with bGH polyA) are: Forward primer 5’- GCA ACT ACC TGG AAC CTG CAA -3’; Reverse primer 5’- GCT GGC AAC TAG AAG GCA CAG T -3’; Probe 5’- AGT AAC CGT CTA GAG GCG -3’. 85-nucleotide sequence from human endogenous SERCA2a cDNA: F5’- TGT GGC CCG CAA CTA CCT -3’; R5’- GCA ACC GAA CAC CCT TAC ATT T -3’; P5’- AAC CTG CAA TAC TGG AGT AA -3’. 143-nucleotide sequence from human endogenous GAPDH: F5’- ATG CTG GCG CTG AGT ACG T -3’; R5’- GTT CAC ACC CAT GAC GAA CAT -3’; P5’- CGT CTT CAC CAC CAT GGA -3’. To construct the DNA standards and serve as a positive control for measuring vector-derived SERCA2a cDNA and mRNA, AAV vector genome has been purified and quantified by qPCR, using the AAV2 reference standard material (VR1616, ATCC). Results are expressed as copy number of single-stranded AAV1/SERCA2a vector per 1000 ng of patient tissue DNA; copy number of single-stranded human endogenous and vector-derived SERCA2a transcripts per 10 ng of patient tissue RNA.

### Laboratory cell and tissue studies

Left ventricular tissue from explanted hearts was cut into 1mm^3^ pieces and washed 4 times for 3 min in 25ml of a low calcium solution (estimated 1-3μM) and gassed with 100% O_2_. Enzymatic digestion was in the same solution supplemented with 50µM CaCl_2_ and containing protease (4(IU)/mL) for 45 min at 35^0^C with gentle shaking.^1^ Two further 45 min digestion periods with 1mg/ml collagenase followed and the cardiomyocytes were collected by centrifugation at 300g for 2 min followed by several washes to remove enzymes. They were then superfused with Krebs-Henseleit solution at 32°C, stimulated at 0.2-1Hz and shortening with each beat quantitated using the IonOptix system.^2^ Late Na^+^ current (I_Na, late_) was evoked and recorded at room temperature (20-22°C) using a similar protocol to that published elsewhere.^3, 4^ The superfusing solution contained (in mM): NaCl 137, CsCl 5.4, glucose 10, HEPES 10, MgCl_2_ 2, CaCl_2_ 1.8, nitrendipine 0.002; pH adjusted to 7.4 (with CsOH). The pipette filling solution contained (in mM): Cs-methane sulfonate 100, NaCl 10, CsCl 30, HEPES 10, EGTA 5, Mg-ATP 5, MgCl_2_ 0.75; pH adjusted to 7.2 (with CsOH).

Cells were depolarized to -20 mV (for 2 s) from a holding potential of -120 mV to evoke Na^+^ current. This depolarizing step was repeated following exposure of the cells to 10 µM ranolazine for 3 min. I_Na, late_ was obtained by subtracting the current recording in the presence of ranolazine from the original recording. I_Na, late_ density was calculated using the average current measured between 210 and 220 ms after the depolarizing step to -20 mV. This time point avoided contaminating the measurement with the early Na^+^ current that completely inactivates within 200 ms.^5^

Human left ventricular 300μm-thick endocardial slices were obtained from transmural tissue samples from one donor heart (n=2 slices), 8 end-stage heart failure transplants (n=14) and 2 failing hearts treated with AAV1/SERCA (n=3). The preparation of ventricular slices was according to the method presented by Camelliti et al.^6^ Slices were electrophysiologically assessed during electrical stimulation using the USB-MEA60-Inv system (MultiChannel Systems, Germany). The MEA consists of 60 titanium electrodes arranged on a 8x8 matrix with 100μm electrode diameter and 700μm intra-electrode distance. A biphasic stimulus (2ms duration; 120% of the threshold voltage, normally 3-4.5V) was applied to obtain 10sec recordings in incremental rates. Cardiomyocyte ultrastructures were visualised by electron microscopy (EM) and tomography (ET) of thin and semi-thick sections, respectively.^7^ Left ventricular tissue fragments were fixed and imaged using an intermediate voltage electron microscope (Tecnai TF30, FEI) operating at 300 kV as described before.

**Supplementary data**

Laboratory cellular and tissue studies

Tissue was obtained from the explanted hearts of the two subjects (3 and 5) who had undergone heart transplantation: both were from the group treated with AAV1/SERCA2a and NAb negative. In the absence of placebo-treated control subjects from within the trial, we performed control experiments on contemporaneously collected myocardial tissue from a donor hearts unsuitable for transplantation after explanation (n=2), and from HF patients with DCM (n=8), ICM (n=3), myocarditis (n=1) and HCM (n=1) whose hearts were explanted for transplantation. Six of these explant patients had received prior LVAD support. No EMB samples were sufficiently large to allow us to perform ex vivo functional studies in addition to the determination of transduction efficiency.

Ventricular cardiomyocytes from the two AAV1/SERCA2a hearts had similar contraction amplitude to those from 8 contemporaneous patients in end stage HF at 0.2Hz, but faster times to 50% or 90% relaxation (see Figure S1A). This effect was sustained when the non-LVAD supported patients were excluded from the control group. Figure S1 shows an example trace from cardiomyocyte isolated from a human heart from patient receiving AAV1/SERCA2a with increasing frequency of stimulation, before and during exposure to the SERCA-inhibitor thapsigargin (20 min pretreatment). The positive effect of increase in frequency on amplitude was evident in three experiments (2.64 ± 1.01% shortening at 0.2Hz to 4.06 ± 0.66% at 1Hz – see Figure S1B). Thapsigargin had a modest effect on amplitude overall, but did not suppress the positive response to frequency (2.34 ± 1.09% at 0.2Hz to 4.77 ± 0.79% at 1Hz). No measurements of SR calcium load were performed. Cardiomyocytes were patch-clamped to measure late sodium current (Figure S1C). While the capacitance of the cardiomyocytes did not vary between HF and AAV1/SERCA2a cardiomyocytes, the late sodium current was lower. Only one of the HF hearts had been LVAD supported: the values for those (0.36, 0.37, 0.29 pA/pF) were closer to the mean (0.33) for the HF group rather than the AAV1/SERCA2a (0.23).

Ultrathin (300μm) slices from the ventricular surface were placed over an MEA and recordings made during pacing from several positions (see Figure S2). There was no apparent difference in field potential duration, but the electrogram amplitude and electrogram in SERCA hearts resembled donor more than failing hearts (see Figure S2E-G).

Blocks were taken from the left ventricle of 5 LVAD patients (two failing human hearts treated with AAV1/SERCA2a and three contemporaneous transplanted failing human hearts) for electron microscopy (EM) and electron tomography (ET) (Fig S3). The main focus was to examine the morphology of the transverse tubule invaginations of the sarcolemmal membrane (T-tubule), as our previous preclinical work has shown normalisation by AAV1/SERCA2a treatment of the disarranged T-tubule morphology in a preclinical HF model.^1^ Interestingly, the physiological predominance of transversely orientated tubules over longitudinal or intermediate elements of the t-tubular system was more apparent in the two AAV1/SERCA2a treated hearts (Transverse:intermediate:longitudinal; 42:25:33 and 42:28:30) compared to HF (30:32:38; 33:32:35 and 31:29:40). Also, the proportion of abnormally dilated t-tubules was also lower in AAV1/SERCA2a hearts (14% and 18%, compared to 21%, 31% and 51% in HF).

**Supplemental figure legends**

Figure S1. Contraction and electrophysiological studies in isolated cardiomyocyte from transplanted human hearts with and without AAV1/SERCA gene delivery.

A. Contraction amplitude (% shortening); time-to-50% (R50) and -90% (R90) relaxation in 25 individual cardiomyocytes from 2 failing hearts treated with AAV.SERCA and 12 cardiomyocytes from 8 failing hearts of patients explanted during the same time period. Median and interquartile range. B. Traces of contraction of an isolated ventricular myocyte from a failing heart treated with AAV1/SERCA before (above) and during (below) exposure to Thapsigargin: a downward deflection represents cell shortening. Frequency of external electrical stimulation was increased as shown. C. Capacitance (pF) and late sodium current in 8 individual cardiomyocytes from 2 failing hearts treated with AAV1/SERCA and 18 cardiomyocytes from 5 failing hearts of patients explanted during the same time period. Median and interquartile range.

Figure S2. Multielectrode array (MEA) recordings from human left ventricular myocardial slices (300 µm deep) from failing hearts including those treated with AAV1/SERCA. Figures 2A-C present data from n=3 slices (derived from 2 AAV1/SERCA2a hearts). Electrogram duration (2A), amplitude (2B) and field potential duration (2C) from AAV1/SERCA2a heart slices is presented with data obtained at cycle lengths of 4000 and 5000ms from two directions of the array (east and north). The stimulation direction did not affect electrogram morphology and there were no changes between pacing rates. Figure 2D presents activation maps obtained from pacing the same ventricular slice from the east side of MEA. Isochronal maps represent a 4x8 electrode area (x and y axes show electrode numbers). The isochrones scale is in msec. Figures 2E-G present data from 2 donor slices (1 heart – black bar), 14 heart failure slices (8 hearts – light grey bar) and 3 AAV1/SERCA2a slices (2 hearts – dark grey bar). These data were obtained at 0.2Hz (cycle length of 5000ms) and the average values from pacing from two directions (east and north) were used for the analysis. The data are expressed as mean+/- S.E.M.

Figure S3. Example low magnification electron micrographs (top row) and high-magnification sliced from 3D electron tomograms from 2 failing human hearts treated with AAV1/SERCA2a and 3 contemporary transplanted failing human hearts (dilated cardiomyopathy, DCM). In the untreated failing hearts, there was a higher prevalence of axial, distorted or distended sarcolemmal tubules (examples indicated with red arrows), whereas in the AAV1/SERCA2a cases the presence of transverse elements (examples indicated with green arrows) was more evident.

Table S5. Troponin I laboratory values (ug/L) at each visit by patient (Reference range: <0.04 ug/L – elevated values highlighted in RED)

| **Subject** | **Treatment** | **Strata** | **Visit BL** | **Visit W1** | **Visit W2** | **Visit W3** | **Visit W4** | **Visit M2** | **Visit M3** | **Visit M4** | **Visit M5** | **Visit M6** |
| --- | --- | --- | --- | --- | --- | --- | --- | --- | --- | --- | --- | --- |
| 1 | AAV1/SERCA2a | AAV  -ve | 0.027 | 0.000 | 0.025 | 0.038 | 0.021 | 0.025 | 0.031 | 0.000 | 0.000 | 0.031 |
| 2 | AAV1/SERCA2a | AAV +ve | 0.000 | 0.000 | 0.000 | 0.000 | ND | 0.000 | UK | 0.000 | 0.000 | ND |
| 3 | AAV1/SERCA2a | AAV  -ve | 0.000 | 0.000 | 0.000 | 0.000 | 0.000 | 0.020 | *0.040* | 0.000 | 0.000 | 0.000 |
| 4 | Placebo | AAV  -ve | 0.027 | *0.048* | *0.058* | *0.072* | *0.059* | *0.049* | *0.042* | ND | *0.046* | 0.033 |
| 5 | AAV1/SERCA2a | AAV  -ve | *0.130* | *0.129* | *0.097* | *0.109* | *0.108* | *0.125* | *0.128* | ND | UK | ND |
|  |  |  |  |  |  |  |  |  |  |  |  |  |
|  |  |  |  |  |  |  |  |  |  | *ND = Not Done | | |
|  |  |  |  |  |  |  |  |  |  | *UK = Unknown | | |

| **Table S6: Exploratory efficacy endpoints – serial measurements and change at 6 months.**  *Delta change at 6 months is from baseline (B), shown as absolute and relative change.*  *Missing data is indicated with – . Subject 5’s clinical deterioration precluded 6WMT assessment at 3 and 6 months and CPEX/study echo assessment at 6 months. CPEX data for subject 2 was incomplete. Calculation of LV ejection fraction was not possible in subjects 1, 2 and 5 due to inadequate apical windows.* | | | | | | | | | | | | | | | |
| --- | --- | --- | --- | --- | --- | --- | --- | --- | --- | --- | --- | --- | --- | --- | --- |
| **Subject** | **Peak VO_2_ (ml/kg/min)** | | | | | **B-type natriuretic peptide (ng/L)** | | | | | **Full speed LV ejection fraction (%)** | | | | |
|  | Serial | | | Delta change at M6 | | Serial | | | Delta change at M6 | | Serial | | | Delta change at M6 | |
|  | B | M3 | M6 | Absolute | Relative (%) | B | M3 | M6 | Absolute | Relative (%) | B | M3 | M6 | Absolute | Relative (%) |
| **1** | 28.3 | 26.9 | 26.6 | -1.7 | -6 | 58 | 101 | 77 | +19 | +33 | - | - | - | - | - |
| **2** | 19.6 | 19.9 | - | - | - | 314 | 343 | 325 | +11 | +4 | - | - | - | - | - |
| **3** | 20.3 | 19.4 | 19.6 | -0.7 | -3 | 37 | 76 | 47 | +10 | +27 | 45 | 40 | 42 | -3 | -7 |
| **4** | 13.5 | 16.4 | 13.9 | +0.4 | +3 | 155 | 88 | 128 | -27 | -17 | 25 | 22 | 26 | +1 | +4 |
| **5** | 15.6 | 9.2 | - | - | - | 359 | 477 | 344 | -15 | -4 | - | - | - | - | - |

| **Subject** | **VE/VCO_2_ slope** | | | | | **6MWD (metres)** | | | | | **LV end diastolic dimension (mm)** | | | | |
| --- | --- | --- | --- | --- | --- | --- | --- | --- | --- | --- | --- | --- | --- | --- | --- |
|  | Serial | | | Delta change at M6 | | Serial | | | Delta change at M6 | | Serial | | | Delta change at M6 | |
|  | B | M3 | M6 | Absolute | Relative (%) | B | M3 | M6 | Absolute | Relative (%) | B | M3 | M6 | Absolute | Relative (%) |
| **1** | 25 | 25 | 27 | +2 | +8 | 623 | 643 | 648 | +25 | +4 | 48 | 54 | 47 | -1 | -2 |
| **2** | 45 | 46 | - | - | - | 627 | 311 | 627 | 0 | 0 | 60 | 61 | 64 | +4 | +6 |
| **3** | 30 | 29 | 29 | -1 | -3 | 480 | 508 | 528 | +48 | +10 | 58 | 59 | 61 | +3 | +5 |
| **4** | 33 | 35 | 34 | +1 | +3 | 563 | 577 | 525 | -38 | -7 | 61 | 56 | 65 | +4 | +7 |
| **5** | 11 | 46 | - | - | - | 397 | - | - | - | - | 58 | 55 | - | - | - |

| Table S7: Detection of SERCA2a transgene in cardiac tissue in clinical trials.  *(IW, inferior wall; LW, lateral wall; AW, anterior wall; AS, anteroseptum; PS, posteroseptum; PW, posterior wall; NSFA, not suitable for analysis; BLD, below limit of detection.)* | | | | | | |
| --- | --- | --- | --- | --- | --- | --- |
| **Clinical Trial** | **Patient ID** | **Dose** | **Months post-infusion** | **Source** | **Heart tissue** | **AAV1/SERCA2a copies DNA per μg of total DNA** |
| **CUPID-1^8^** |  | 1.4x 10^11^ | 8 | Tx | AS, PS, AW, PLW | BLD |
|  |  | 3x 10^12^ | 1  21 | LVAD  Tx | LVAC  AS, PS, AW, PLW | BLD  BLD |
|  |  | 3x 10^12^ | 5 | Tx | AS, PS, AW, and PLW | BLD |
|  |  | 3x 10^12^ | 10 | Tx | AS, PS, AW, and PLW | BLD |
|  |  | 3x 10^12^ | 11 | LVAD | LVAC | BLD |
|  |  | 1x 10^13^ | 18 | Biopsy | PLW | BLD |
|  |  | 1x 10^13^ | 11  23 | LVAD  Tx | LVAC  AS  PS  AW, PLW  LVAC  RVAC | >20 to <200  561  365  >20 to <200  230  250 |
|  |  | 1x 10^13^ | 31 | Unspec | LVAC | >20 to <200 |
|  |  | 1x 10^13^ | 22 | Tx | PLW  AS, PS, AW | 223  >20 to <200 |
| **CUPID-2^9^** |  | 1x 10^13^ | 12 | LVAD | LVAC | 14-62 |
|  |  | 1x 10^13^ | 13 | Tx | AS  PS  AW  PLW | 80  71  36  77 |
|  |  | 1x 10^13^ | 1.5 | Tx | AS  PS  AW  PLW  LVAC  RVAC | 26  37  40  134  <10  <10 |
|  |  | 1x 10^13^ | 8 | Tx | AW  Heart | 115  72-123 |
|  |  | 1x 10^13^ | 14 | Tx | AS  PS  AW  PLW | 27  84  62  102 |
|  |  | 1x 10^13^ | 10 | Tx | AS  PS  AW  PLW  LVAC | 43  33  53  37  36 |
|  |  | 1x 10^13^ | 29 | Tx | Heart | 192 |
| **SERCA-LVAD** | 003 | 1x 10^13^ | 6 | Biopsy | IW, LW | NSFA |
|  | 004 | 1x 10^13^ | 6  22 | Biopsy  Tx | IW(a)  IW(b), LW  AW, AS, PS, PW | 38  BLD  BLD |
|  | 007 | 1x 10^13^ | 6 | Tx | AW  AS  PS  PW | 41  80  57  23 |

1. Ideally the respiratory exchange ratio (RER) should be <0.7 before starting exercise. In this patient group this is usually not achieved, and in these cases suboptimal RER would have to be accepted. [↑](#footnote-ref-1)
2. This can prevent inappropriate therapies during the test that can precipitate collapse and falling off the treadmill [↑](#footnote-ref-2)
