# Supplementary figures and images for: Investigation of the safety and feasibility of AAV1/SERCA2a gene transfer in patients with chronic heart failure supported with a left ventricular assist device – the SERCA-LVAD TRIAL
Source: Gene Ther. 2020 Jul 15;27(12):579–90. doi: 10.1038/s41434-020-0171-7 (PMC7744277; doi:10.1038/s41434-020-0171-7)

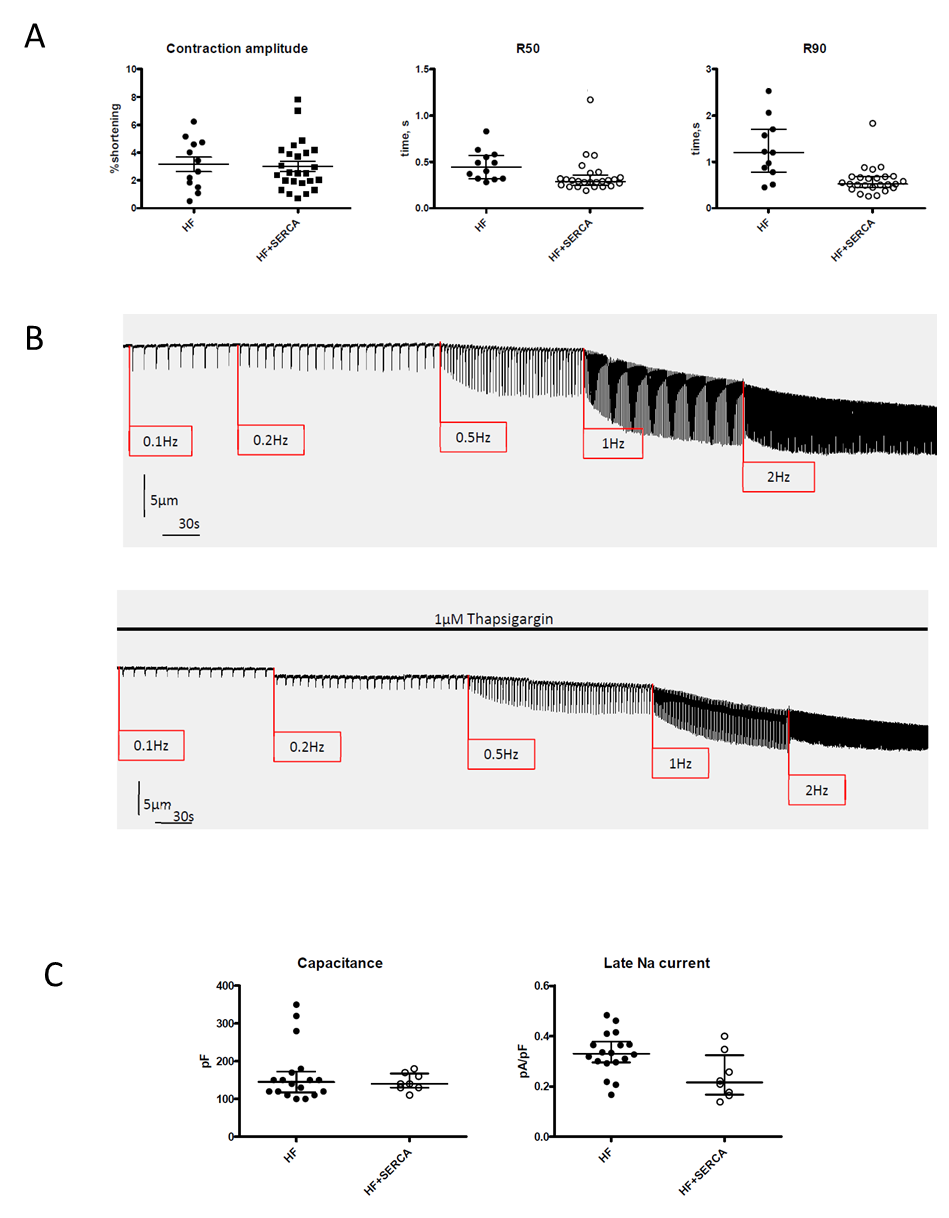

Supplement: Supplementary file 2 — Figure S1 [file 41434_2020_171_MOESM2_ESM.tif]

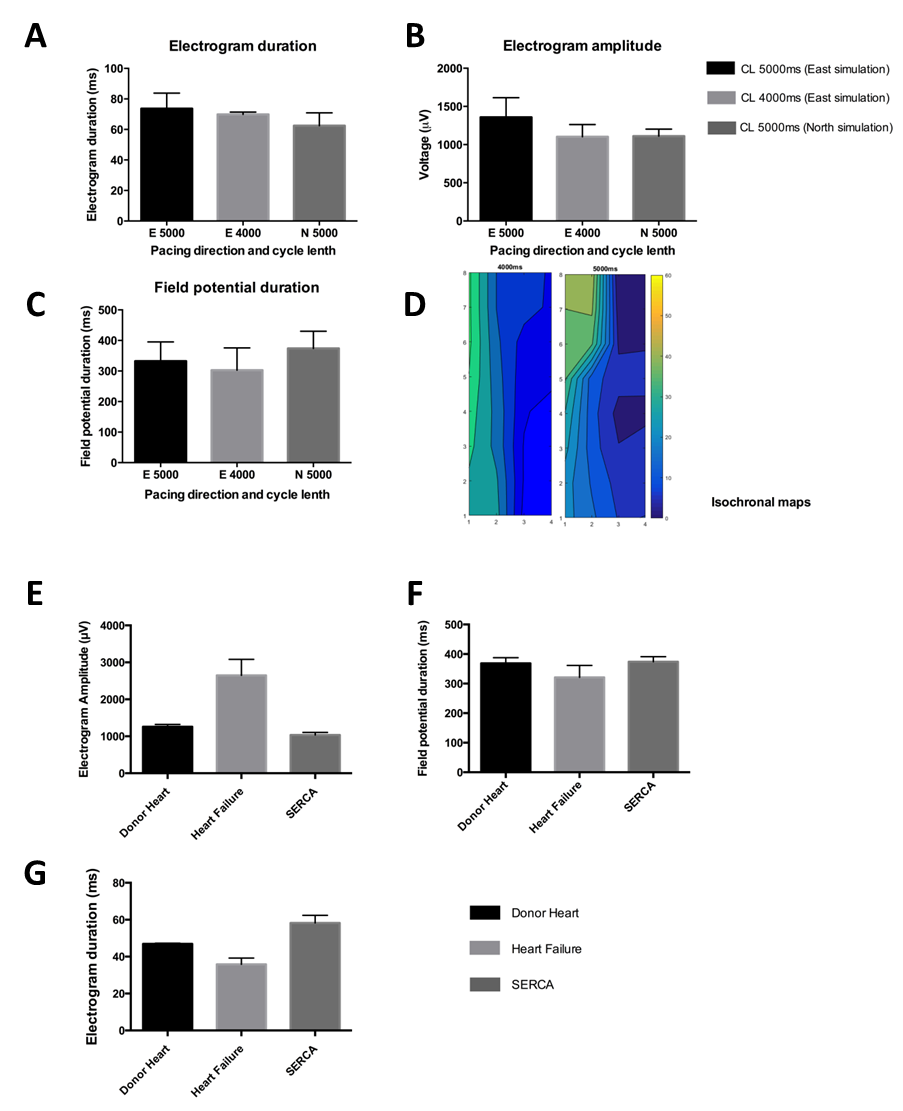

Supplement: Supplementary file 3 — Figure S2 [file 41434_2020_171_MOESM3_ESM.tif]

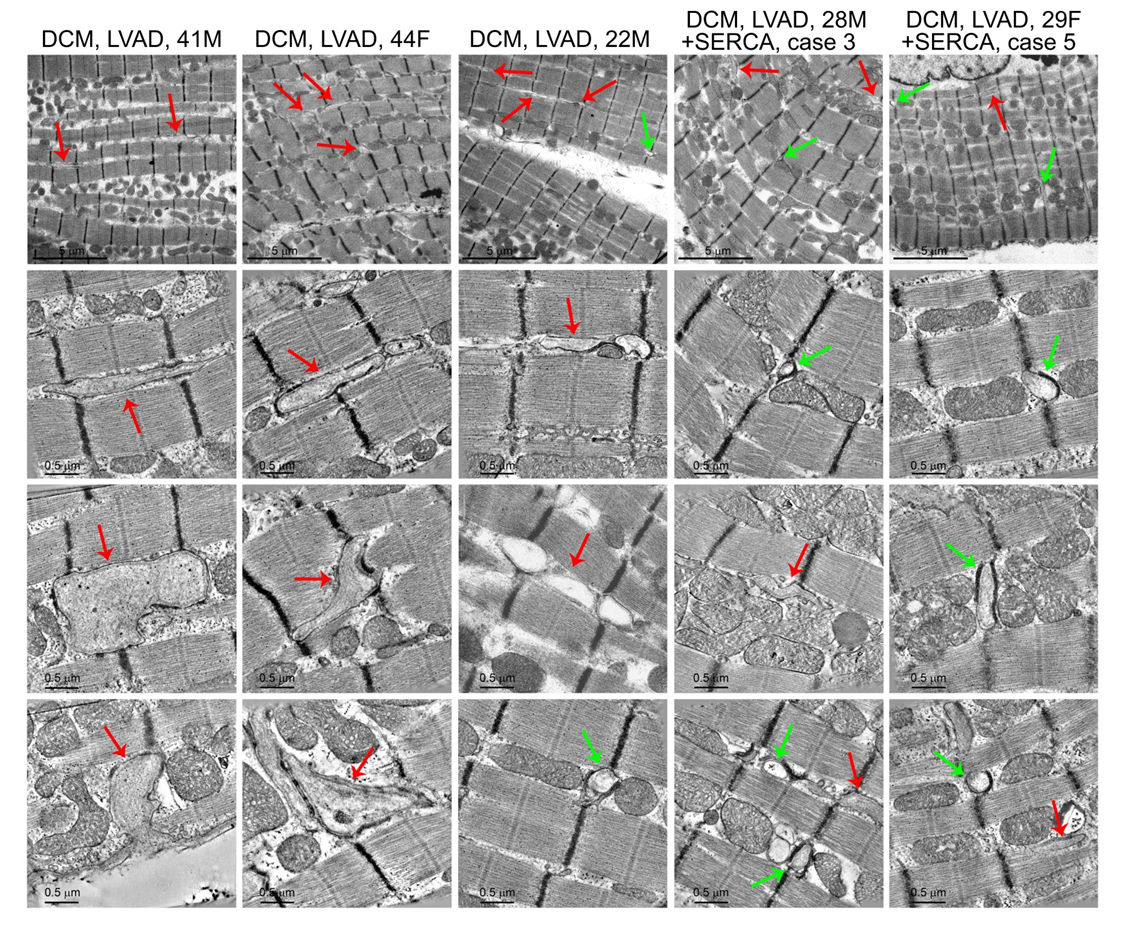

Supplement: Supplementary file 4 — Figure S3 [file 41434_2020_171_MOESM4_ESM.tif]
